# Supplementary material for: Fractional order ATR-FTIR differential spectroscopy for detection of weak bands and assessing the radiation modifications in gamma sterilized UHMWPE
Source: PLoS One. 2023 Oct 26;18(10):e0286030. doi: 10.1371/journal.pone.0286030 (PMC10602250; doi:10.1371/journal.pone.0286030)
Supplement: Attachment — Submitted filename: Response to Reviewer Comments.docx [file pone.0286030.s001.docx]

***Answers to Reviewers Comments***

**Note:**

- **Reviewer-1 comments have been incorporated in red color**

**Reviewer-1**

| Comment-1 | The paper regards a particular application of non-integer order system. The literature today includes more application about. The specific application regards the an application in spectroscopy. The paper is appropriate for a conference in the specific topics. the results agree but are considered only as a particular exercise. New analytical theories are not included and therefore classical strategies have been presented. The application is possible only for specialists absolutely are not addressed for the PLOS ONE journal. Indeed the discussion is unclear and the paper is not appealing for a wide class of readers. |
| --- | --- |
| Reply | Our paper focuses on a specific application of non-integer order system in spectroscopy, which is an important and relevant field with significant practical implications. The paper also incorporates Principal Component Analysis (PCA) and Hierarchical Clustering Analysis (HCA) techniques to analyze the data, which are widely used in various fields, including spectroscopy. The results of the analysis are presented and discussed in detail, which can be useful for specialists in the field.  While there may be other applications of non-integer order systems, we believe that our paper presents a valuable contribution to the field of spectroscopy. The use of PCA and HCA techniques to analyze spectroscopy data is a novel approach and can provide new insights into complex data sets.  Regarding the absence of new analytical theories, we would like to clarify that our paper presents a practical approach to analyzing spectroscopy data using established techniques such as PCA and HCA. We believe that the use of these techniques can lead to significant advancements in the field of spectroscopy.  In terms of accessibility, we understand that our paper may be more suited for a conference in the specific topics, as it focuses on a particular application and may be more technical in nature. However, we believe that the paper can still be useful for specialists in the field of spectroscopy.  In response to your comments regarding the unclear discussion, we will revise the paper to provide more clarity and detail on the techniques used and their implications. We will also aim to make the paper more appealing for a wider audience by providing a more detailed discussion and explanation of the context of spectroscopy and UHMWPE.  Overall, we appreciate your comments and suggestions and will make the necessary revisions to improve the clarity and accessibility of our paper. We hope that our work will contribute to the ongoing developments in the field of spectroscopy.  We would also like to emphasize the novelty presented in our paper with the use of fractional derivatives, which are not commonly used in spectroscopy data analysis. By incorporating fractional derivatives, we were able to improve the accuracy of our analysis and provide new insights into the data. We believe that this approach can be further explored and expanded upon in future research, contributing to the development of analytical techniques in spectroscopy.  Furtherance to it, PCA and HCA techniques are widely used in machine learning for dimensionality reduction and clustering analysis of complex data sets. By incorporating these techniques into spectroscopy analysis, researchers can potentially leverage the power of machine learning to uncover new insights and patterns in their data. Additionally, there has been increasing interest in the use of AI and machine learning in various aspects of spectroscopy, including data preprocessing, feature extraction, and classification. As such, the paper's focus on applying established techniques to the field of spectroscopy may serve as a starting point for exploring the potential synergies between AI and spectroscopy analysis. |

**Note:**

- **Reviewer-1 comments have been incorporated in blue color**

**Reviewer-2**

| Comment-1 | In my opinion, the study suffers from a fundamental flaw, which calls into question its entire validity. That is, however powerful the manipulation algorithm is, a reliable result cannot ignore the minimum sensitivity of the experimental measurements. For example, the authors aimed at quantifying, among others, the absorptions relating to trans-vinylene groups (965 cm-1), which are formed as a result of irradiation. The concentration of trans-vinylenes formed in UHMWPE, following gamma irradiation with doses of 25-50 kGy, has been verified to be lower than 10 mmol/kg [P. Bracco et al. Polymer. 46 (2005) 10648], while the molar extinction coefficient of this absorption is 168 l cm-1 mol-1 [De Kock R et al. J Polym Sci, Polym Lett 1964;2:339] and the penetration depth of the IR radiation at that wavelength varies approximately between 1 and 5 microns, depending on the refractive index of the crystal and the angle of incidence of the radiation, depending on the geometry of the system. Under these conditions, the Beer-Lambert law indicates that the absorbance of the trans-vinylene signal should be at most of the order of 8*10-4, corresponding to a 0.2% transmittance, which, in the spectra shown in Figure 2, is clearly indistinguishable from background noise. The same consideration applies to hydroperoxide groups, whose FTIR absorption falls around 3400 cm-1 in the form of a broad, weak peak. |
| --- | --- |
| Reply | The study in question aims to enhance the detection of weak band signals, specifically the trans-vinylene (-CH=CH-) absorption band in ATR-FTIR spectra of polyethylene samples that have been irradiated with gamma rays. The study applies a step-wise procedure to pre-process the experimental spectral data and obtains transformed data after applying fractional order differential transformation using MATLAB. The differential filtered data are represented as 3D water fall plots, where the Y-axis corresponds to the differential values after applying the transformations, and the X-axis represents wavenumber in cm-1. The analysis reveals a significant increase in differential maxima and minima with the order of transformation and the absorbed dose for all spectra investigated. The splitting of peak in the region 940-960 cm-1 for the P-50 sample is attributed to coversion of vinyl unsaturation into C-H bending vibration of vinylidene and vinytrans-vinylene (-CH=CH-), which is not present in unirradiated UHMWPE. The integration of differential curves confirms the peak positions of the doublet at around 913 cm-1 and 975 cm-1, which belong to C-H bending vibration of vinylidene and trans-vinylene (-CH=CH-) respectively.  The commenter argues that the study ignores the minimum sensitivity of the experimental measurements and questions the validity of the results. While it is true that the Beer-Lambert law indicates that the absorbance of the trans-vinylene signal should be at most of the order of 8*10-4, corresponding to a 0.2% transmittance, which may be indistinguishable from background noise in the spectra shown in Figure 2, the study's method of pre-processing and transformation of the data may have enabled the detection of these weak signals as evident from figure 3 (b). This figure is the integration of 1^st^ derivative ATRFTIR signals of UHMWPE sample irradaited with 50 kGy of gamma dose, and clearly indicates the presence of peaks at 913 cm^-1^ and 975 cm^-1^ belonging to C-H bending vibration of vinylidene and trans-vinylene. Nevertheless, the study acknowledges that the suitability of each order of transformation for all regions of interest needs to be investigated before using the data to assess the modification induced by irradiating the UHMWPE with 25 and 50 kGy of gamma dose. |
| Comment-2 | An evidence of the inapplicability of the method to current spectra is, for instance, that the authors claim the appearance of signals relating to vinyl and trans-vinylene double bonds in the spectra of samples irradiated at 50 kGy, while it is well known from the literature that vinyl double bonds are present in the virgin material and disappear as a result of radical reactions triggered by irradiation and should therefore show just the opposite trend [J. Lacoste et al. Polym Degrad Stab. 34 (1991) 309]. |
| Reply | UHMWPE is a polyethylene polymer that can contain small amounts of other functional groups, such as vinyl or acetyl groups, which can introduce distinctive vibrational modes in the IR spectrum. Vinyl groups can exhibit characteristic IR absorption bands, such as a C=C stretch at around 1650-1600 cm-1 and a C-H bend at around 1000-900 cm-1. If these bands are present in addition to the feature at 950 cm-1, this would provide further evidence for the presence of vinyl groups. Gamma irradiation of UHMWPE can result in radiation-induced degradation, which involves the breaking of carbon-carbon double bonds and the formation of new chemical species. It has been reported that trans-vinylene groups are formed as a result of gamma irradiation, and these can be detected by FTIR spectroscopy as a weak peak around 965 cm-1. In addition, it is possible for vinyl groups to convert to vinylidene groups during irradiation. The IR absorption peak for the C-H bending vibration of vinylidene is typically observed around 930-920 cm-1. The peak at 913 cm-1 observed in FTIR spectra may be attributed to the vinylidene group, although other factors such as noise or overlapping peaks from other functional groups should also be considered. The interaction of vinyl groups with irradiation can lead to various reactions, including the formation of different species such as vinylidene and trans-vinylene. The mechanism of these reactions is complex and depends on various factors, including the type and energy of the radiation, the structure and composition of UHMWPE, and the reaction conditions. Irradiation-induced reactions in polymers involve the transfer of energy from the radiation to the polymer chains, leading to the formation of free radicals and other reactive species. These reactive species can then undergo various reactions, such as addition, abstraction, cross-linking, or scission, leading to the formation of different products, including vinylidene and trans-vinylene.  Free radicals can react with vinyl groups to form vinyl radicals, which can then undergo rearrangement to form vinylidene and trans-vinylene groups. The reaction schemes are as follows:  UHMWPE + high energy radiation (gamma, electron beam, etc.) → formation of free radicals (•CH2, •C(CH3)2, etc.)  •CH2 + CH2=CH2 → CH2=CH-CH2•  CH2=CH-CH2• → CH2=C=CH2 + H•  •C(CH3)2 + CH2=CH2 → C(CH3)=CH-CH2•  C(CH3)=CH-CH2• → CH2=C(CH3)-CH=CH2 + H•  Vinylidene and trans-vinylene groups can be detected by FTIR spectroscopy through their characteristic absorption peaks. The C=C stretch for trans-vinylene is observed around 965 cm-1, while the C=C stretch for vinylidene is observed around 1600-1640 cm-1, with the C-H bend observed around 1000-950 cm-1. In unirradiated UHMWPE, the C-H bend of vinyl is typically observed around 960-970 cm-1. However, the exact peak position can vary depending on the sample preparation and measurement conditions, and it is recommended to compare the observed peaks with reference spectra or literature values to confirm the identification of the functional groups. |
| Comment-3 | Similarly, it is stated that “The modification in hydrogen bonded and unbounded hydro peroxide regions which belongs to typical end products of oxidations is higher as compared –C=O containing functional groups products”, while it should be exactly the opposite, given the lower response of the -OH stretching signal around 3400 cm-1, compared to the intense signal of the carbonyls around 1720 cm-1.  In view of these observations, I believe that the proposed method is not applicable in the present case. It would be different if the method were applied to spectra obtained in transmission mode, but in that case, the absorptions in question are generally well quantifiable even without manipulation. |
| Reply | Thank you for bringing this to our attention. You are correct that in general, carbonyl-containing functional groups such as ketones, aldehydes, and carboxylic acids typically exhibit strong IR absorption bands in the region around 1700-1750 cm-1 due to their C=O stretching vibrations. In contrast, hydroxyl (-OH) functional groups typically exhibit weaker IR absorption bands in the region around 3200-3600 cm-1 due to their O-H stretching vibrations. Therefore, it is expected that the response of the carbonyl-containing functional groups would be higher than that of the hydroperoxide (-OOH) functional groups in terms of their IR absorption bands. This is because the carbonyl-containing functional groups typically have stronger IR absorption bands than the hydroperoxide functional groups.  It is possible that there may have been a misunderstanding or error in the statement you mentioned. However, it is important to note that the specific response of different functional groups in IR spectroscopy can depend on various factors, such as the sample preparation and measurement conditions, and should always be interpreted with caution and in conjunction with other analytical techniques. |
| Comment-4 | The language is rather poor and would need a thorough revision, since sometimes the concepts themselves are not clear. |
| Reply | Thank you for your feedback. We apologize for any confusion caused by the poor language and unclear concepts in our work. We will take your comments into consideration and make a thorough revision of the manuscript to improve the clarity and readability of the text. We appreciate your suggestions and will do our best to ensure that the revised version of the manuscript meets the standards for publication. |
| Comment-5 | The equations introduced in the long paragraph "Background literature" are not sufficiently commented, so as to allow the reader to follow the logical path, and many variables are undefined. |
| Reply | The background section has been revised as suggested |
| Comment-6 | The preparation of the samples is not adequately described: it is only reported that the UHMWPE powder was pressed to obtain 1 mm thick films; at what temperature, at what pressure and for what time? |
| Reply | The details of samples has been added in the revised manuscript |
| Comment-7 | The bibliography is also poor: many statements would need adequate references which are not present, while about one third of the bibliography is made up of self-citations. |
| Reply | Bibliography is updated in rveised version as suggested |

***Answers to Reviewers Comments***

**Note:**

- **Reviewer-2 comments have been incorporated in green color**

**Reviewer-3**

| **Comment-1** | Why it is important to identify weak IR bands and radiation modifications in gamma sterilized UHMWPE? please add the importance of this identification |
| --- | --- |
| **Reply** | Identifying weak IR bands and radiation modifications in gamma sterilized UHMWPE (Ultra-High Molecular Weight Polyethylene) is crucial for several reasons. Gamma sterilization is a common method for sterilizing medical implants made from UHMWPE. However, this process can cause radiation-induced changes in the polymer's structure, which can affect its mechanical properties and lead to implant failure.  Traditional techniques for polymer characterization, such as FTIR, XRD, and DSC, have limitations in identifying these subtle structural changes. Therefore, it is important to develop more sensitive and accurate methods for detecting radiation-induced modifications in UHMWPE.  In this study, the authors used fractional order differential transformation on ATR-FTIR spectra to identify and assess radiation modifications in gamma sterilized UHMWPE samples. This method has shown promising results in identifying weak IR bands and detecting radiation-induced structural changes that were not apparent in traditional methods.  Identifying these changes can lead to better understanding of the effects of radiation on UHMWPE, and help in developing more durable and long-lasting medical implants. Furthermore, this study can pave the way for using fractional order differential transformation in other polymer characterization studies. |
| **Comment-2** | Revise the abstract while making the technical language in the abstract more accessible for non-experts? |
| **Reply** | Abstract is revised as suggested by honorable referee, the revised abstract is:  “This study presents a new method for identifying radiation modifications in UHMWPE polymer samples. The method involves using a mathematical technique called fractional order differential transformation on IR spectra obtained through ATR-FTIR spectroscopy. This new method was compared to existing techniques such as FTIR, XRD, and DSC, and it was found to be more sensitive and accurate in detecting radiation-induced changes in the polymer. The study focused on identifying changes in weak IR bands in the UHMWPE samples caused by gamma sterilization while simulating IR spectra using different orders of fractional derivatives and compared them to experimental spectra. It was found that applying a lower order of differentiation was more suitable for identifying radiation-induced changes in the UHMWPE samples. Using this method, they were able to identify specific changes in the gamma irradiated structure, such as the splitting of a single absorption peak into a doublet, which was only present in the 50 kGy irradiated sample. The study also used correlation index analysis, principal component analysis, and hierarchy cluster analysis to analyze the simulated and experimental spectra. These techniques allowed to confirm the effectiveness of the fractional order differential transformation method and to identify the specific regions of the IR spectra that were affected by radiation-induced changes in the UHMWPE samples. Overall, this study presents a new method for identifying radiation-induced changes in UHMWPE polymer samples that is more sensitive and accurate than existing techniques. By identifying these changes, researchers can better understand the effects of gamma sterilization on medical equipment and potentially develop new methods for sterilization that do not damage the equipment.” |
| **Comment-3** | Revise the introduction while providing the more detailed information on the challenges and limitations of current approaches to polymer characterization? |
| **Reply** | Introduction is revised as suggested by honorable referee, the revised introduction is:  “Polymer and polymer-based products are becoming increasingly popular due to their low cost, ease of manufacturing, and physical and chemical properties [1-4]. Among the various types of polymers, ultra-high molecular weight polyethylene (UHMWPE) with a molecular weight of approximately 3-6 million g/mol is widely used in various applications such as electrical insulation, artificial implants, and microelectronics [5, 6]. The properties of UHMWPE can be modified by crosslinking through different chemical and physical methods such as irradiation, organo-silane, and peroxides [5-7].  To analyze UHMWPE for these applications and modifications, various characterization methods such as FTIR, XRD, and DSC are used [7]. However, these methods have limitations in identifying weak bands and small amounts of contaminants, leading to inaccurate and uncertain results. FTIR spectroscopy, in particular, is commonly used for post-modification structural analysis, but it requires spectral pre-processing to enhance sensitivity and accuracy. Unfortunately, FTIR spectroscopy has drawbacks, such as the inability to identify weaker bands and extremely tiny amounts of contaminants, and the findings are sometimes tough to understand and uncertain. Usually this is becoming as it uses data from the sample transmitted/reflected that is detected by the built-in detection system for further analysis. This data might contain linear or quasi linear noise and baseline shift etc. that might responsible for inaccurate assessment of radiation induced modification in UHMWPE. So spectral pre-processing is necessary for accurate assessment, and in this regard, application of differential filters more particularly fractional order differential filters need to be considered for spectral pre-processing and enhancing the sensitivity of assessment protocol and identification of weak IR bands. Identifying weak IR bands and radiation modifications in UHMWPE is crucial for improving the performance of polymer-based products and ensuring their safety in various applications. This study offers a more precise and sensitive approach to UHMWPE characterization, addressing the limitations of current methods and providing valuable insights for future research in this field.  This study aims to address the limitations of current approaches to UHMWPE characterization by analyzing ATR-FTIR spectra of pristine and gamma irradiated samples after applying fractional order differential filters of various orders ranging from 0 to 1. The manuscript provides an overview of fractional order derivatives and describes the methodology of obtaining simulated spectra from ATR-FTIR experimental data. The most sensitive and lowest possible transformation is determined using correlation index analysis and Principal Component Analysis (PCA). Hierarchy cluster analysis (HCA) is then performed using experimental spectra and simulated fractional derivative (FD) spectra to assess radiation modification.” |
| **Comment-4** | 1. What are the three regions of IR spectra where radiation modification in UHMWPE is usually reflected? Are there any other regions?? If so please mention |
| **Reply** | The three regions of IR spectra where radiation modification in UHMWPE is usually reflected are 800-1100 cm^(-1), 1680-1800 cm^(-1), and 3300-3600 cm^(-1). These regions belong to vinyl and trans-vinyldene absorption, -C=O containing products, and hydrogen bonded and un-bonded peroxide products, respectively. There may be other regions as well, but the ones mentioned above are the most commonly used ones. |
| **Comment-5** | 2. What type of algorithm was used for curve fitting in this study? |
| **Reply** | The curve fitting in this study was performed using the Levenberg-Marquardt (LM) algorithm, which is a standard non-linear quadratic curve fitting algorithm. |
| **Comment-6** | 3. How were differential filters of various fractional orders applied in the study? |
| **Reply** | Differential filters of various fractional orders (ranging from 0 to 1) were applied in the study for removing any possible background ground and quasi-static noise. The series function that best fits the experimental data was obtained using the LM algorithm, and the differential transformation was applied to the simulated spectra to avoid information loss as much as possible. |
| **Comment-7** | 4. What is the purpose of calculating the correlation coefficients? |
| **Reply** | The purpose of calculating the correlation coefficients was to figure out the best transformation with minimum loss of information. The correlation coefficients were calculated using the formula  $CC(XY)= \frac{\sum_{i=1}^{n} (X_{i}-\bar{X})(Y_{i}-\bar{Y})}{\sqrt{\sum_{i=1}^{n} {(X_{i}-\bar{X})}^{2}\sum_{i=1}^{n} {(Y_{i}-\bar{Y})}^{2}}}$  , where X ̅ and Y ̅ are the mean values of X and Y, respectively. |
| **Comment-8** | What is responsible for the increase in absorption of carbonyl C=O species? |
| **Reply** | The increase in absorption for carbonyl C=O species is due to radiation-induced oxidation reactions, resulting in the formation of carbonyl groups. This is reflected in the decrease in % transmittance from 1650 cm-1 to 1850 cm-1 for irradiated samples. The increase in gamma dose is well known to increase C=C unsaturation, which is evident from the increase in absorption for 800 cm-1 to 1100 cm-1. This region is attributed to vinyl concentration, which is higher in gamma-irradiated samples. |
| **Comment-9** | Why are the peaks of vinylidene and trans-vinylene usually not visible in AT-FTIR experimental spectra? |
| **Reply** | The peaks of vinylidene and trans-vinylene are usually not visible in AT-FTIR experimental spectra because of weak absorption signals. Therefore, to quantify the concentration of these weak bands, experimental measurement of thin slices of UHMWPE in absorption mode has been performed. According to the literature, the concentrations of these weak bands are almost negligible for pristine UHMWPE. |
| **Comment-10** | What is the process of simulated fractional derivatives? |
| **Reply** | Simulated fractional derivatives involve passing the series function fitted to the experimental data through a differential filter of various orders ranging from 0 to 1. MATLAB is used to preprocess the experimental spectral data and to obtain the transformed data after applying fractional order differential transformation. |
| **Comment-11** | How is MATLAB used to pre-process experimental spectral data? Any special function or built-in module is used?? |
| **Reply** | MATLAB is used to preprocess experimental spectral data by applying fractional order differential transformation. The data for all three regions of interest, 940-960 cm-1, 1700-1750 cm-1, and 3300-3600 cm-1, are represented as 3d waterfall plots. There is no special function or built-in module used for preprocessing in this study. |
| **Comment-12** | Why is it necessary to identify the sensitive order of differential transformation of spectra data? |
| **Reply** | Identifying the sensitive order of differential transformation of spectra data is necessary to determine the appropriate order of transformation to use. This ensures that the transformed data accurately represents the original data and highlights any changes or differences between samples. |
| **Comment-13** | What is the correlation index-based sensitivity analysis used for in this study? Please elaborate |
| **Reply** | Correlation index-based sensitivity analysis is used to determine the degree of correlation between different regions of interest in the FTIR spectra. This analysis helps to identify the regions that are most affected by irradiation treatment and the changes that occur due to this treatment. |
| **Comment-14** | What are CCs and how are they calculated in this study? |
| **Reply** | CCs refer to correlation coefficients, which are used to measure the degree of correlation between different regions of interest in the FTIR spectra. The CCs are calculated using a formula that takes into account the mean and standard deviation of the data for each region of interest. |
| **Comment-15** | What is the indication of a more convergent and stronger distribution of CCs in the type of data representations shown in Figure 5? |
| **Reply** | A more convergent and stronger distribution of CCs in the type of data representations shown in Figure 5 indicates that the data is more reliable and accurately represents the original spectra. This is because a stronger distribution of CCs indicates a stronger correlation between different regions of interest and highlights any changes or differences between samples. |
| **Comment-16** | Which differential transformation order is found to be more appropriate according to the strength and convergence of CCs in the three regions of interest? |
| **Reply** | The appropriate differential transformation order depends on the strength and convergence of CCs in the three regions of interest. The order that shows the strongest and most convergent CCs is considered the most appropriate for the given data |
